# Gut Microbiome Alterations and Functional Prediction in Chronic Spontaneous Urticaria Patients

**DOI:** 10.4014/jmb.2012.12022

**Published:** 2021-03-19

**Authors:** Xinyue Zhang, Jun Zhang, Zhaowei Chu, Linjing Shi, Songmei Geng, Kun Guo

**Affiliations:** 1 Department of Dermatology, The Second Affiliated Hospital, School of Medicine, Xi'an Jiaotong University, Xi'an 710004, P.R. China; 2 College of Science, Northwest University, Xi’an 710069, P.R. China

**Keywords:** Gut microbiome, urticaria, dysbiosis, bacterial diversity

## Abstract

The effects of the gut microbiome on both allergy and autoimmunity in dermatological diseases have been indicated in several recent studies. Chronic spontaneous urticaria (CSU) is a disease involving allergy and autoimmunity, and there is no report detailing the role of microbiota alterations in its development. This study was performed to identify the fecal microbial composition of CSU patients and investigate the different compositions and potential genetic functions on the fecal microbiota between CSU patients and normal controls. The gut microbiota of CSU patients and healthy individuals were obtained by 16s rRNA massive sequencing. Gut microbiota diversity and composition were compared, and bioinformatics analysis of the differences was performed. The gut microbiota composition results showed that Firmicutes, Bacteroidetes, Proteobacteria, and Verrucomicrobia were dominant microbiota in CSU patients. The differential analysis showed that relative abundance of the Proteobacteria (*p* = 0.03), Bacilli (*p* = 0.04), Enterobacterales (*p* = 0.03), *Enterobacteriaceae* (*p* = 0.03) was significantly increased in CSU patients. In contrast, the relative abundance of *Megamonas*, *Megasphaera*, and *Dialister* (all *p* < 0.05) in these patients significantly decreased compared with healthy controls. The different microbiological compositions impacted normal gastrointestinal functions based on function prediction, resulting in abnormal pathways, including transport and metabolism. We found CSU patients exhibited gut microbiota dysbiosis compared with healthy controls. Our results indicated CSU is associated with gut microbiota dysbiosis and pointed out that the bacterial taxa increased in CSU patients, which might be involved in the pathogenesis of CSU. These results provided clues for future microbial-based therapies on CSU.

## Introduction

Chronic urticaria (CU) is one of the most common skin diseases with high incidence, as roughly 15-25% of the population have suffered from it [1]. CU comprises both chronic spontaneous urticaria (CSU) and chronic inducible urticaria (CIndU), which could be triggered by cold, hot, pressure, etc. [2]. CSU is a type of CU that requires excluding other types for diagnosis, and 50% of CSU patients respond poorly to antihistamine therapy [3]. Recently, multiple studies have suggested that autoimmunity (IgG-mediated disease) and autoallergy (IgE-mediated disease) can contribute to the pathogenesis of the CSU and predispose subjects to the development of other autoimmune diseases [4]. CU, especially CSU, deserves further study and research, whether as a traditional allergic disease or a systemic immune disease.

The microbiome lives on or inside another organism, and most of these microorganisms inhabit the human gut [5]. In recent years, it has been reported that the microbiome, which is influenced by multiple environmental and dietary factors, could be an essential factor in health or disease by modulating the immune response and other pathophysiology processes [6]. Significantly, gut microbiota dysbiosis is associated with chronic inflammatory disorders of the skin, such as atopic dermatitis (AD) and psoriasis [7-13]. It has been reported that gut microbiota may contribute to the development, persistence, and severity of AD via immunologic, metabolic, and neuroendocrine pathways [8, 14]. Besides, S. Manfredo found that a gut pathobiont could translocate and promote autoimmunity in lupus-prone mice, antibiotic treatment, or intramuscular vaccination against *Enterococcus gallinarum*-suppressed autoimmune manifestations, which transform microbiome study from pathogenesis to treatment [15]. The above information suggests that the microbiota is involved in the occurrence and development of these diseases and can be used as a new therapeutic target to control them.

Previous work on the association between CU and microbiota focused on changing relative amounts of a few common microbiotas [16, 17]. They found the relative amounts of *Akkermansia muciniphila*, *Clostridium leptum*, *Faecalibacterium prausnitzii*, *Lactobacillus*, and *Bifidobacterium* were different in fecal samples from CU patients compared to normal controls. The latest research showed that the microbial composition was significantly different between CU patients and controls; they pointed out that *Escherichia coli* is a pathogenic strain in CU [18]. These results implicate the change of microbiota in patients with CU; however, gut microbiota dysbiosis in CU was restricted by the number of samples and lack of repeats, especially the deficiency of research focusing on CSU, so further studies are required to confirm the findings. So far, there has been no comprehensive study on compositional and structural changes of the gut microbiota in people with CSU. In the current research, we first utilized novel high-throughput DNA sequencing and showed that patients with CSU possessed a gut microbiota dysbiosis compared with normal controls in community composition and predicted gene functions. Our results indicated that CSU was associated with compositional and structural changes in the gut microbiota. We will attempt to explore how this potential link affects the occurrence and development of CSU, modulating the cutaneous immune response in urticarial and other allergic diseases, and finally, how to take advantage of microbiota in further therapy.

## Materials and Methods

### Study Population

A total of 20 patients with a clinical diagnosis of CSU (CSU is defined as the presence of urticaria for over six weeks and is distinct from the inducible urticaria) in our clinic at the Second Affiliated Hospital of Xi’an Jiaotong University from July 2018 to December 2018, along with 20 healthy, age- and sex-matched individuals, were included in the present study. Exclusion criteria included antibiotic usage during one month before the study, infectious disease, allergic and autoimmune diseases, and cancer in both groups. The study’s protocol was approved by the local Ethical Committee of the Second Affiliated Hospital of Xi’an Jiaotong University. All volunteers received information concerning their participation in the research and gave written informed consent.

### Fecal Sample Collection, DNA Extraction, and Sequencing

Approximately 2 g of fecal samples was collected in a sterile plastic cup and stored at -80°C immediately. According to manufacture protocols, microbial DNA was extracted using the HiPure Stool DNA Kit (China). The purity of extracted DNA was confirmed via spectrophotometric analyses (A260/280 ratio of 1.8) using a NanoDrop ND-1000 Spectrophotometer (Nucliber). All extracted DNA samples were stored at -20°C until further analysis.

The amplification of the variable region V3-V4 of the 16S rRNA gene was performed to analyze the samples' microbial populations. PCR was conducted using the bacterial universal primers 341F: CCTACGGGNGGC WGCAG and 806R: GGACTACHVGGGTATCTAAT. All PCR reactions were performed in triplicate using a 50-μl mixture containing 5 μl of 10× KOD Buffer, 5 μl of 2.5 mM dNTPs, 1.5 μl of each primer (5 μM), 1 μl of KOD polymerase, and 100 ng of template DNA. Briefly, after an initial denaturation step at 95°C for 2 min, the amplifications were conducted with 27 cycles at a melting temperature of 98°C for 10 s, an annealing temperature of 62°C for 30 s, and an extension temperature of 68°C for 30 s. The final extension step was performed at 68°C for 10 min. Amplicons were recovered from 2% agarose gels and purified using the AxyPrep DNA Gel Extraction Kit (Axygen Biosciences, USA) according to the manufacturer’s instructions and quantified using a QuantiFluor-ST (Promega, USA). The purified amplicons were pooled in equimolar, and sequencing was performed by Genedenovo Inc. (China) on the Illumina Hiseq 2500 PE250 (paired-end sequenced) platform (Illumina, USA) according to the standard protocols.

### Processing of Sequencing Data

Raw data obtained after sequencing included dirty reads containing adapters or low-quality bases, which would affect the following assembly and analysis. Thus, to get high-quality clean reads, raw reads were further filtered according to the following rules: 1) Removing reads containing more than 10% of unknown nucleotides (N); 2) removing reads containing less than 80% of bases with quality (Q-value) > 20. The filtered reads were then assembled into tags according to overlap between paired-end reads with overlap of more than 10 bp and less than 2% mismatch. The software Mothur (v.1.34.0) [19] was used to remove the redundant tags to get unique tags. The obtained unique tags were then used to calculate the abundance. The software rdp classifier (http://rdp.cme.msu.edu/classifier/classifier.jsp) was used to classify tags into different taxonomies against the Greengenes database [20] (version 20101006) with a confidence threshold of 0.8.

Mothur was again used to cluster tags of more than 97% identity into OTUs, and the abundances of OTUs were calculated. The taxonomic classification of OTUs was based on the annotation result of contained tags according to the mode principle; that is, the taxonomic rank, which included more than 66% of tags, was thought to be the taxonomic rank of this OTU; otherwise, the higher rank would be considered. The taxonomic ranks in descending order of size are domain, phylum, class, order, family, genus, and species.

The software Metastats was used to detect the differentially abundant microbial community between two samples, and the FDR value was used to evaluate difference significance. Based on the result of Metastats analysis, the significant differential species were filtered by |Log2 (FC)| ≥1 and *p*-value ≤ 0.05 to do pathway enrichment analysis.

### Microbial Gene Function Prediction and Analysis

The gene function profiles of stool microbiota were predicted with PICRUSt, which forecasts gene abundances of metabolic functions based on the 16S copy number- corrected OTU composition. Functional genes were categorized (by PICRUSt) into Clusters of KEGG Orthology (KO) gene families. To identify gene functions of different bacterial communities between CU patients and healthy controls, the abundances of Clusters of Orthologous Groups (COGs) families were scaled by total sum per sample and subjected to enrichment analysis of two-group comparison using the Wilcoxon signed-rank test.

### Availability of Data and Materials

The datasets generated for this study can be found in the SRA accession database: ID PRJNA650123.

## Results

### Participants

A total of 40 participants, including 20 with CSU (6 males, 14 females) and 20 healthy controls (6 males, 14 females), were recruited for this study. The CSU group and control group were matched for gender ((male: 30% vs. 30%, respectively, *p* = 1.00) and age (42.2 ± 3.1 vs. 41.95 ± 2.9, respectively, *p* = 0.9532) (Table 1).

### Operational Taxonomic Unit (OTU) Annotation

The DNA sequencing results from 40 samples obtained through filtering, with an average of 114,236 tags, were measured per sample. After assembling and abundance statistics, 42,711 unique tags were obtained on average.

**Fig. 1:**
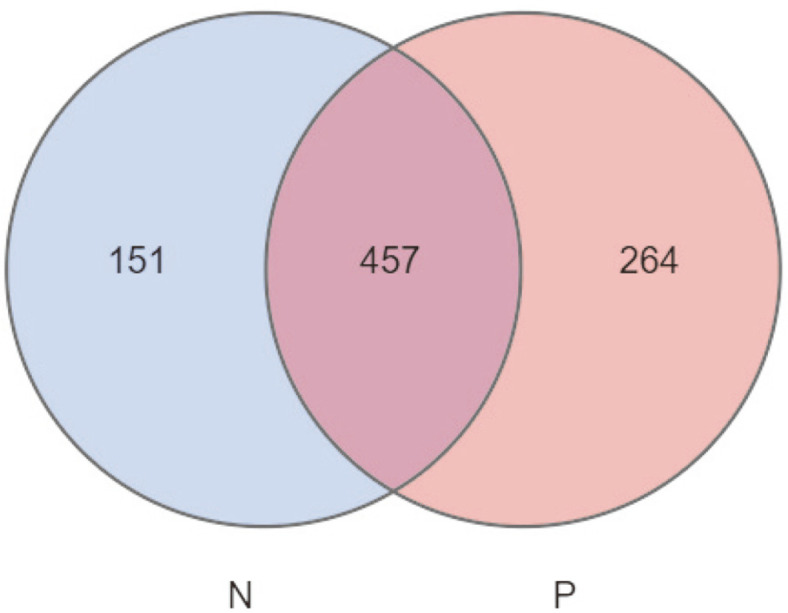
Venn diagram showing the unique and shared operational taxonomic units (OTUs) in the healthy control group (N) in green colored and the CSU patient group (P) in red colored.

**Fig. 2:**
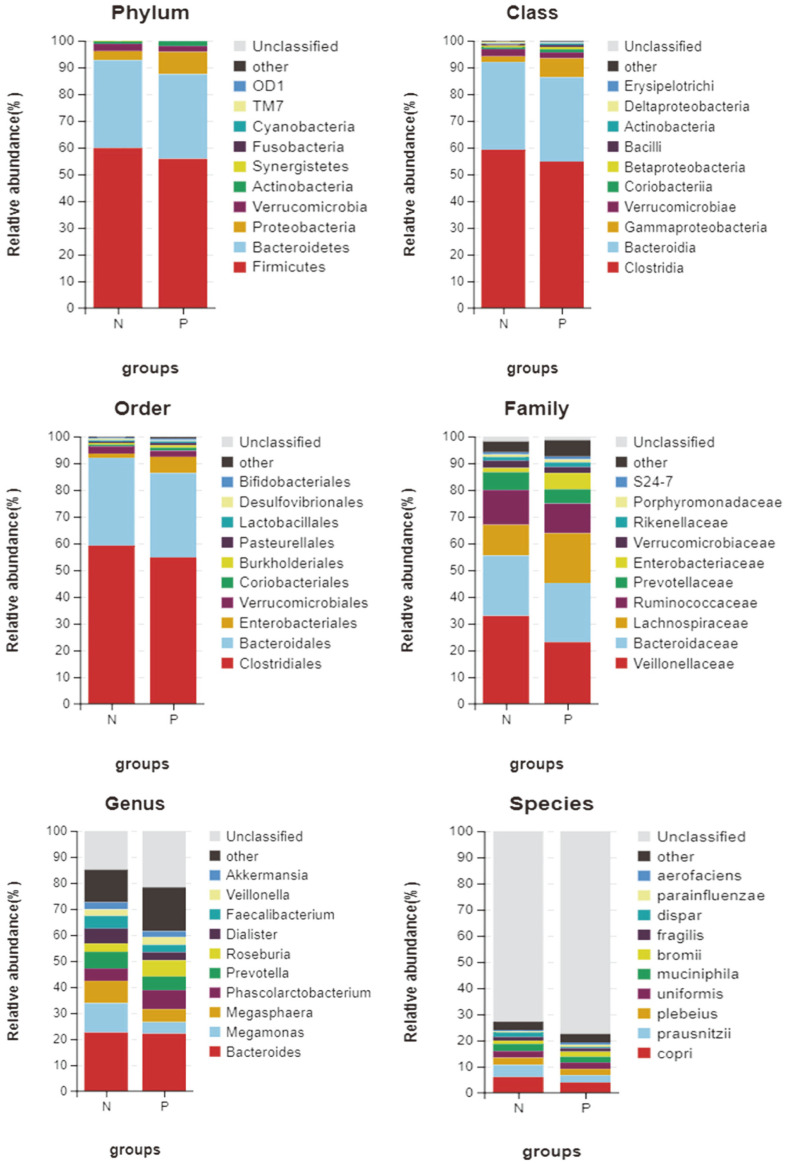
The composition of gut microbiota in CSU patients group (P) and healthy controls group (N). The top 10 taxa in relative abundance at different levels.

After clustering tags of more than 97% identity into OTUs, we collected a total of 872 OTUs. We used Venn diagrams to visually display the number of shared and unique OTUs in different groups. There were 457 shared OTUs in both groups, with 151 unique OTUs in the normal control group (N) and 264 unique OTUs in the CSU group (P). The results indicated that 75.16% (457/608) and 63.38% (457/721) of the common OTUs were identified in these two groups (Fig. 1). Then, OTUs were annotated against the KEGG database. We found that the top 10 taxa in relative abundance at the phylum level include: Firmicutes, Bacteroidetes, Proteobacteria, Verrucomicrobia, Actinobacteria, Synergistetes, Fusobacteria, Cyanobacteria, TM7, and OD1; the top 10 taxa in relative abundance at the class level include: Clostridia, Bacteroidia, Gammaproteobacteria, Verrucomicrobiae, Coriobacteriia, Betaproteobacteria, Bacilli, Actinobacteria, Deltaproteobacteria, and Erysipelotrichi; the top 10 taxa in relative abundance at the order level include: Clostridiales, Bacteroidales, Enterobacteriales, Verrucomicrobiales, Coriobacteriales, Burkholderiales, Pasteurellales, Lactobacillales, Desulfovibrionales, and Bifidobacteriales; the top 10 taxa in relative abundance at the family level include: *Veillonellaceae, Bacteroidaceae, Lachnospiraceae, Ruminococcaceae, Prevotellaceae, Enterobacteriaceae, Verrucomicrobiaceae, Rikenellaceae, Porphyromonadaceae*, and S24-7; the top 10 taxa in relative abundance at the genus level include: *Bacteroides, Megamonas, Megasphaera, Phascolarctobacterium, Prevotella, Roseburia, Dialister, Faecalibacterium, Veillonella*, and *Akkermansia*; the top 10 taxa in relative abundance at the species level include: *copri, prausnitzii, plebeius, uniformis, muciniphila, bromii, fragilis, dispar, parainfluenzae*, and *aerofaciens* (Fig. 2).

### Bacterial Diversity Analysis

**Fig. 3:**
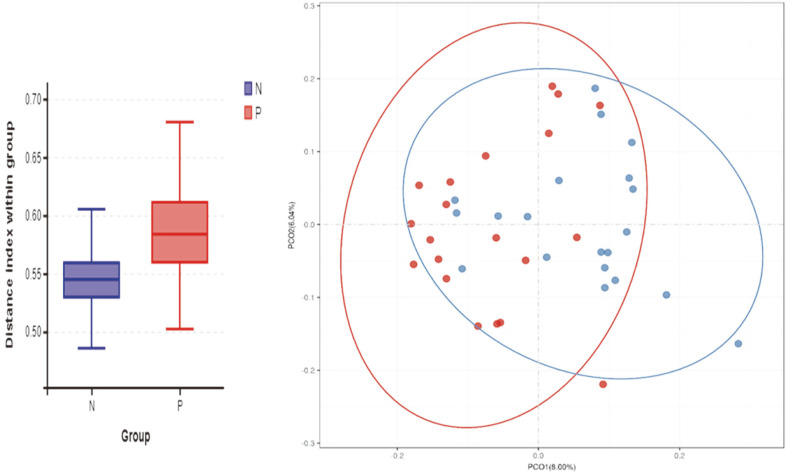
Analysis of beta diversity revealed significant differences between CSU patients group (P) and healthy controls group (N) performed on the unweighted UniFrac (*p* < 0.001). The results revealed a significant separation in the bacterial community composition between CSU patients and healthy individuals. PCoA scatter diagram based on unweighted Unifrac data between samples. In the analysis results, the more similar the samples are, the closer the distances reflected in the PCoA diagram are. Red dots stand for patients group (P) and blue dots stand for controls group (N). It revealed significant clustering of patients group (P) and controls group (N).

The alpha diversity represented species diversity in a single sample and was evaluated by several diversity indices such as chao1 value, ACE value, Shannon index, and Simpson index [21], etc. Compared with the normal control group (N), the patient group (P) had no significant difference in these indexes, including ACE, Shannon, Sobs, Chao, and Simpson, based on the Wilcoxon test (Supplement 1 and Supplement table). This result indicated no significant microbial diversity in the patient group (P) than the normal control group (N). Unlike alpha diversity, beta diversity is the ratio of all OTUs, and common OTUs between two groups are often used to determine the differentiation among groups. Beta analyses performed on the unweighted UniFrac distance matrix and represented through box graph and principal coordinates analysis (PCoa) revealed significant clustering (*p* < 0.001) of healthy controls (N) and patients (P), supporting the results that the gut microbiota composition differed between both groups (Fig. 3).

### Community Structure Differences

**Fig. 4:**
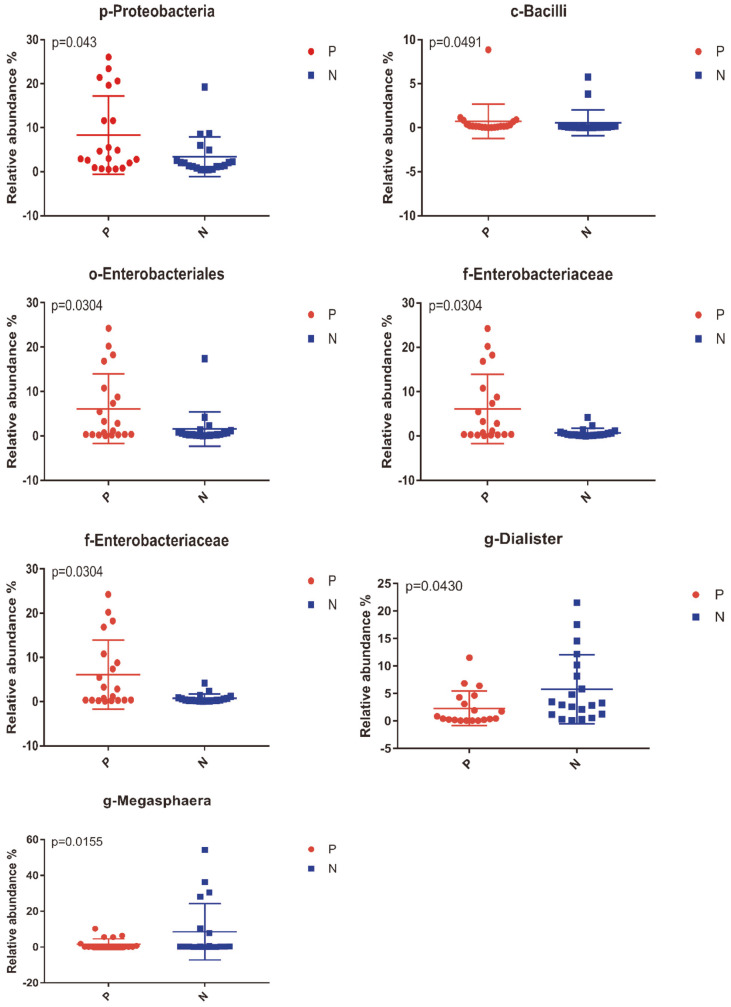
Relative abundance at different bacterial levels between CSU patients (P) and controls (N) groups was compared. The taxa with significant differences (*p* < 0.05) were shown including Proteobacteria at phylum level, Bacilli at class level, Enterobacterales at order level, *Enterobacteriaceae* at family level, *Megamonas*, *Dialister* and *Megasphaera* at genus level.

We further analyzed the composition of the intestinal microflora of the patient group (P) and normal controls (N). According to the taxonomic classification and abundance of OTUs, each group's abundance on each taxonomic level by domain, phylum, class order, family, genus, and species was calculated. Moreover, we compared the different abundances of one species in different groups to find out the significantly different species among patients and normal controls. At the phylum level, the patient group displayed a significant increase in Proteobacteria (*p* = 0.03). Bacilli were increased significantly in the patient group at the class level compared with controls (*p* = 0.04). At the order level, patients displayed a significant increase in Enterobacterales (*p* = 0.03). At the family level, *Enterobacteriaceae* was relatively more abundant in the patient group (*p* = 0.03). At the genus level, *Megamonas*, *Megasphaera*, and *Dialister* were relatively less abundant in patients (all *p* < 0.05) (Fig. 4).

### Bacterial Gene Functions

**Fig. 5:**
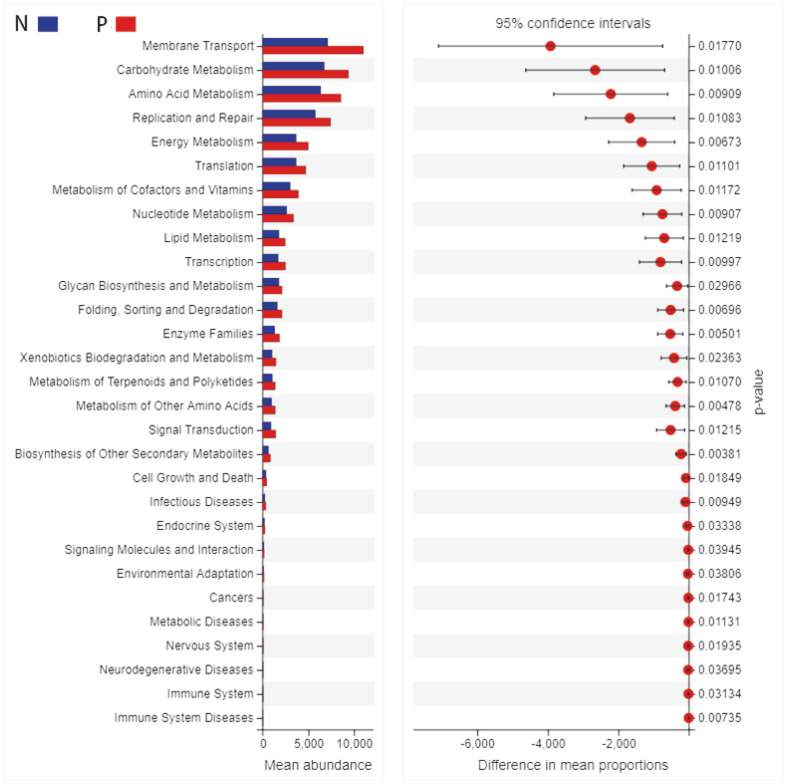
Differential pathway functional analysis between patients group (P) in red and controls group (N) in blue. Left side: the ordinate is the different KEGG pathway, and the abscissa represents the abundance of the different pathway. Right side: the abscissa is the confidence interval range of the abundance difference between groups, and the ordinate is the *p*-value.

With the development of analytical technology, community function prediction using diversity sequencing data has become an essential part of microbial research. Based on the community structure derived from the Greengenes database, the functional profile of different groups was predicted from referenced bacterial genomes by PICRUSt [22]. According to OTU abundance information in the database, PICRUSt can annotate the KEGG Pathway functions and count the abundance information of each pathway to draw the statistical chart of KEGG database prediction results (Fig. 5). We screened the pathway with a statistically significant difference between the patient group (P) and controls group (N). Metabolic enzymes involved in carbohydrate metabolism, amino acid metabolism, energy metabolism, and metabolism of cofactors and vitamins and nucleotide metabolism were more abundant in patients’ fecal microbiota.

## Discussion

In this study, the composition of the gut microbiota of the 20 patients with CSU was elucidated based on 16S rRNA gene profiling to evaluate the potential bacterial dysbiosis compared with a healthy control group of 20 individuals from the same geographic location. Due to the lack of previous studies in this field, we can only combine the possible pathogenesis of CSU with microbiota dysbiosis in our results to infer the corresponding conclusions, providing clues for subsequent studies.

There was no statistically significant difference between the CSU patients and normal healthy people in alpha diversity, demonstrating that the levels of community species diversity and richness were similar in these two groups. Previous studies on bacterial diversity pointed out that bacterial diversity increased allergic sensitization risk [23], but the correlation between bacterial diversity and disease is still controversial. Several studies on psoriasis have shown lower microbial diversity in psoriasis patients, and others found no difference in bacterial diversity in patients than normal controls [9, 24]. The clustering methods and statistical methods used in each study are different, leading to inconsistent conclusions. Results were not consistent as the sample size changed, so the relationship between bacterial diversity and etiology of CSU remains to be further studied.

In contrast to alpha diversity used in diversity within a sample, beta diversity is used to describe diversity between different samples and indicate the similarity of two samples. We found that beta diversity revealed significant differences between the CSU group and the healthy control group. The two groups were able to be clustered independently in the PCOA diagram. This result confirmed microbiota dysbiosis in CSU patients, and then we analyzed the specific differences of the microbial community at different levels.

Studies on gut microbiota composition have shown that Bacteroidetes and Firmicutes are dominant microbiota in adults, whereas Actinobacteria, Proteobacteria, and Verrucomicrobia, although found in many people [25, 26]. Our results were consistent with these studies and showed that these common gut flora detected in healthy adults also appeared in CSU patients. Besides, we found the relative abundance of Proteobacteria increased significantly in CSU patients compared with controls. Although Proteobacteria are common bacteria found in the human gut, changes in their relative abundance could also cause microbiota dysbiosis, even related to diseases. It was reported that Proteobacteria were increased in patients with asthma and allergic diseases [27, 28], and they also explained that Proteobacteria might cause allergies by upregulating Th17-related genes. Increased Th17 cells were detected in patients with chronic urticaria compared with normal controls, suggesting that the occurrence of chronic urticaria may also be closely related to Th17 cells [29]. Another study found that Proteobacteria was enriched in HIV-positive patients, and they thought Proteobacteria and Firmicutes could be used as biomarkers in the HIV infection screening test [30]. Also, the abundance of Proteobacteria can be altered by the action of drugs; for example, highly active antiretroviral therapy (HAART) appeared to reduce them in HIV-positive patients, and atorvastatin showed relatively increased levels of Proteobacteria in high-fat diet rats and patients with atherosclerosis [31]. We suppose that more mechanism studies will support that Proteobacteria are related to the occurrence of chronic urticaria. In that case, as a kind of intestinal flora regulated by drugs, Proteobacteria will be an excellent therapeutic target.

Many pathogenic bacteria, including *E. coli* and *Salmonella*, were classified into the Proteobacteria phylum [32]. Though we could not find the significant differences in *E. coli* and *Salmonella* between CSU patients and controls, we found Bacilli were increased in CSU patients at the class level. Many genera of probiotic bacteria pertained to gram-positive, non-spore-forming Bacilli such as *Lactobacillus*; however, gram-negative Bacilli could play a significant role as a pathogen in infection. According to a review on chronic urticarial pathogenesis, the infection could be an important trigger in CSU [33]. So, increased Bacilli in CSU patients suggested that there might be certain infections related to CSU in patients' intestinal tracts. We also found that the relative abundance of Gammaproteobacteria in CSU patients were higher than in control, but the difference was not significant (*p* = 0.07). However, at the order level, the Enterobacterales belonging to Gammaproteobacteria were significantly increased (*p* < 0.05). The *Enterobacteriaceae* family of Gammaproteobacteria were elevated in mucosal or stool samples from adenoma cases compared to controls. The common conclusion from several studies was that *Enterobacteriaceae* elevated in adenoma patients compared with healthy controls [34], and they regarded *Enterobacteriaceae* as potential colorectal cancer driver bacteria. The possible mechanism was that *Enterobacteriaceae* could cause chronic recurrent inflammation in the gastrointestinal tract [35, 36].

Our previous discussion focused on the microbiota significantly elevated in CSU patients. We also found some reduced levels of bacteria in patients. *Megamonas*, *Megasphaera*, and *Dialister* were reduced considerably in CSU patients at the genus level than controls. The correlation analysis between gut microbiota of HSP and the clinical indices showed that the level of serum IgE was negatively associated with *Megamonas* [37]. It was reported that IgE antibodies played a vital role in the relationship between chronic spontaneous urticaria and autoimmune diseases [38]. According to the clinical characteristics of chronic urticaria, the serum IgE level in the patient group was higher than control, which verified the negative correlation between the abundance of *Megamonas* and serum IgE level. *Megasphaera* and *Dialister* were related to a person’s weight, and both of them tended to show the same trend in different studies [39, 40]. This result indicated that the enrolled population's body weight (or BMI) should be considered in case-control studies related to intestinal microorganisms to avoid sampling bias.

The dominant bacterial species largely determine the function of the gut microbiota community. It is known that metagenomic sequencing aims to sample all genes from a community and can produce detailed metabolic and functional profiles. But as a preliminary exploratory study, deep metagenomic sequencing across many samples was exceedingly expensive for this study. For now, we used PICRUSt (phylogenetic investigation of communities by reconstruction of unobserved states) as a computational approach to predict the functional composition of a metagenome using marker gene data and a database of reference genomes. The principle and scientificity of PICRUSt are described in the previous study, and this approach has been used widely for microbial research [41]. Previous studies reported that gut microbiota could affect human immune response through structural modification of intestinal mucosa, endocrine, metabolic mechanisms, and neurological transmission [42]. Our function prediction results showed that the differences between the patients and the control group were mainly in the membrane transport and metabolic pathway. According to the pathogenesis of CSU, we assumed metabolic products corresponding to abnormal gut microbiota might enter the blood as quorum-sensing molecules (QSMs) through the interaction with Mas-related G-protein coupled receptor member X2 (MRGPRX2) to activate mast cells and promote the occurrence of urticaria [43]. As for increased nutrient metabolism in CSU patients, this could be explained by the fact that those increased bacteria may promote the metabolism of carbohydrates, amino acids, and energy. In the next step, metagenomics can further extend and verify the 16s rRNA sequencing analysis results, and metabolomics analysis can be used to find specific differential metabolites associated with gut microbiota. The mechanism of gut microbiota dysbiosis in CSU will soon be revealed through the existing techniques and methods.

In conclusion, we preliminarily described the elemental gut microbiota composition in CSU patients by sequencing the gut microbiota in 20 CSU patients' fecal samples. We then compared the gut microbiota of 20 normal controls and found significant differences in the relative abundance of florae in CSU patients, especially Proteobacteria, Bacilli, and Enterobacterales, which were abnormal in other diseases. Further functional prediction confirmed that these differential microbiota compositions could lead to dysfunction, including metabolites. Our study pointed out the gut microbiota dysbiosis in CSU and predicted that gut microbiota might influence CSU's pathogenesis through metabolic abnormalities. Further studies are warranted to identify meaningful microbial biomarkers and therapeutic targets for CSU.

## Figures and Tables

**Table 1:** Information about chronic urticarial patients (P) and healthy controls (N).

Index	Group P	Group N	P
Mean age, years	42.2±3.1	41.95±2.9	0.9532
Sex (M/F)	6:14	6:14	1.00
BMI	22.44 ± 2.807	22.16 ± 2.263	0.6733
